# Brief pharmacist-led intervention using a structured questionnaire to improve attitudes toward medication adherence in patients on antipsychotics: a single-site before–after study

**DOI:** 10.1186/s12991-026-00636-7

**Published:** 2026-03-03

**Authors:** Masaki Maehara, Masayasu Sugiyama, Daisuke Kobayashi, Junpei Mutoh, Kanta Noguchi, Mitsuhiro Wada

**Affiliations:** 1 Sugiyama Pharmacy Co., Ltd., 525-2 Mitsusawa, Ogoori, 838-0106 Fukuoka Japan; 2 Graduate School of Pharmaceutical Sciences, Sanyo-Onoda City University, 1-1-1 Daigakudori, Sanyo-Onoda, Yamaguchi, 756-0884 Japan; 3 Department of Clinical Pharmacy and Pharmaceutical Care, Graduate School of Pharmaceutical Science, Kyushu University, Fukuoka, 812-8582 Japan; 4 Faculty of Pharmaceutical Sciences, Sanyo-Onoda City University, 1-1-1 Daigakudori, Sanyo-Onoda, Yamaguchi, 756-0884 Japan

**Keywords:** Medication adherence, Pharmacist intervention, Antipsychotics, Brief intervention, Drug attitude inventory-10, Psychiatric patients

## Abstract

**Background:**

Medication adherence among patients with psychiatric disorders is a significant challenge that often leads to poor clinical outcomes. Although pharmacist-led interventions have demonstrated the potential to improve medication adherence, evidence remains limited, particularly in patients treated with antipsychotics. This study aimed to evaluate the effectiveness of a brief pharmacist-led intervention in enhancing medication adherence among outpatients receiving antipsychotic therapy.

**Methods:**

This exploratory, prospective, single-arm study was conducted at a community pharmacy in Japan. Fifty outpatients prescribed antipsychotic medications participated in the study. A brief pharmacist-led intervention was delivered focusing on identifying and addressing barriers to adherence. The primary outcomes were changes in Drug Attitude Inventory-10 (DAI-10) scores and the proportion of participants with unused medications.

**Results:**

Participants taking preexisting medications exhibited a significant improvement in DAI-10 scores (mean increase, + 2.05 points; from 5.81 ± 4.00 to 7.86 ± 2.90, *p* = 0.00619). The proportion of participants who reported non-adherence to medication regimens decreased significantly from 42% to 24% (*p*= 0.0159).

**Conclusions:**

The brief pharmacist-led intervention was effective in improving attitudes toward medication and behavioral adherence among patients receiving antipsychotic treatment in our study group. These findings underscore the importance of active pharmacist engagement for supporting medication adherence.

## Background

Severe mental disorders, including schizophrenia, major depressive disorder, and bipolar disorder, are chronic and recurrent conditions that require sustained pharmacological treatment [1]. Adherence to medication is a critical determinant of treatment success. Discontinuation of antipsychotic therapy is one of the primary factors associated with relapse, and repeated relapses may contribute to subsequent treatment-resistance in a subset of patients [2, 3]. Furthermore, treatment satisfaction and medication preferences have been linked to improved adherence [4], highlighting the need for active monitoring and patient-centered support in pharmacotherapy.

In Japan, psychiatric care has been undergoing a transition from hospital-centered to community-based services in line with governmental policies aimed at promoting deinstitutionalization [5]. This shift toward community-based psychiatric care is consistent with the global mental health policies promoted by the World Health Organization, which emphasize deinstitutionalization and continuity of care in community settings [6]. Consequently, community pharmacies are increasingly expected to play an expanding role in mental health care by not only dispensing medications but also offering counseling, monitoring treatment adherence, and identifying drug-related problems in collaboration with other healthcare professionals [7, 8].

However, pharmacists often encounter challenges in effectively communicating with patients with severe mental disorders, partly because of persistent stigma [9, 10]. This communication barrier may impede the detection of medication-related problems such as poor medication adherence, dissatisfaction with therapy, or adverse drug events, thereby limiting the effectiveness of community pharmacy-based interventions.

Several previous interventions aimed to improve medication adherence through patient education on illness, environmental support, and telephone-based nursing interventions [11]. Pharmacist-led approaches, including medication counseling and adherence support, have demonstrated positive effects on adherence-related outcomes [11–13]. In addition, pharmacist-led interventions have been shown to improve medication attitudes among hospitalized patients, as assessed using the Drug Attitude Inventory-10 (DAI-10), among hospitalized patients [14].

Previous studies, including ours, have demonstrated that incorporating structured questionnaires into medication counseling may facilitate the identification of drug-related problems and improve patient engagement [7, 14]. Nevertheless, previous interventions primarily focused on adverse events and did not adequately address medication regimen-related issues such as dosing frequency and timing, which are crucial determinants of medication adherence. Although emerging evidence suggests that pharmacist-led interventions may improve patient medication attitudes and medication-taking behaviors [15], data focusing on community settings remain scarce.

We hypothesized that a brief pharmacist-led intervention would improve attitudes and adherence behaviors among patients taking antipsychotics. Accordingly, this study aimed to evaluate the effectiveness of a brief pharmacist-led intervention using a structured questionnaire that focused on medication regimen problems in a Japanese community pharmacy setting. Although similar pharmacist-led adherence interventions have been reported in other countries [15], to the best of our knowledge, this is the first study of Japanese community pharmacies to investigate the impact of such intervention.

## Methods

### Study design and setting

We conducted an exploratory, before–after, observational, mirror-image study at a community pharmacy located near a psychiatric hospital in Japan between June 1, 2023 and December 31, 2023.

In Japan, outpatient prescriptions are typically issued by physicians and dispensed at community pharmacies; many patients regularly obtain their medications from the same pharmacy, allowing pharmacists to have repeated contact with patients.

### Participants

Eligible participants were adults (aged ≥ 18 years) who were prescribed at least one antipsychotic medication at the time of enrollment. Representatives who collected prescriptions on behalf of patients were excluded unless they were actively involved in supporting the patient’s medication adherence. Written informed consent was obtained from all participants after they were provided with detailed information about the study, including its purpose, procedures, confidentiality, and voluntary participation. In addition, outpatient prescriptions dispensed at community pharmacies in Japan do not routinely include psychiatric diagnoses. Therefore, diagnostic information could not be systematically collected in this study and participants were defined based on antipsychotic prescriptions rather than specific diagnoses.

### Procedures

Before receiving baseline medication counseling, the participants completed Questionnaire A (assessing medication-related problems) and the DAI-10 [16]. The pharmacists then conducted individualized counseling at a private counter. If the participants consented, the pharmacists provided a brief feedback report (“tracing report”) to their prescribing physicians summarizing the identified medication-related issues.

During the second visit, pharmacists reinforced medication education, addressed questions, and explained the potential adverse effects of the modified medication regimens. At the third visit, the participants completed Questionnaire A and the DAI-10.

The intervention followed a standardized framework consisting of a structured assessment using Questionnaire A and pharmacist-led counseling. The specific content of counseling was individualized based on each patient’s identified medication-related problems. Throughout the study period, all counseling sessions and follow-up assessments were conducted by the same pharmacist, who had prior clinical experience in psychiatric pharmacotherapy, to ensure consistency of the intervention and outcome assessment.

### Brief intervention

The intervention primarily included (1) education on strategies for managing missed doses; (2) explanation of meal effects on the pharmacokinetics of medication; (3) counseling about adverse effects and alternative treatment options for prescribed medication; and (4) individualized education on illness insight and medication literacy tailored to each patient’s specific needs.

### Questionnaire A

Questionnaire A was a structured, pharmacist-developed questionnaire designed to identify medication-related problems. It assessed medication-taking behaviors, including the frequency and timing of missed doses, type of poor adherence (intentional vs. unintentional), difficulties with medication administration, experience of adverse events, and an open-ended section for participants to describe any other problems or preferences regarding their medication. Changes in responses before and after the intervention were descriptively compared to evaluate improvements in medication-related problems.

### Data collection

Baseline demographic and clinical data (sex, age, duration of antipsychotic use, and medication history) were extracted from pharmacy records. Questionnaire A and DAI-10 scores were collected at baseline and postintervention.

### Outcomes

The primary outcomes were the change in patients’ attitudes toward medication adherence, as assessed using the DAI-10 scores, and the change in the proportion of patients who did not use antipsychotic medications at baseline and after the intervention. The DAI-10 consists of 10 items, yielding a total score ranging from − 10 to + 10. Positive scores indicated a favorable attitude toward medication adherence, whereas negative scores indicated an unfavorable attitude. Secondary outcomes were specific reasons for non-adherence, including intentional and unintentional factors.

Information regarding unused medication was obtained through patient self-reports using Questionnaire A. Unused medication was defined as regularly prescribed medication that was not taken as directed; medication prescribed on an as-needed basis was excluded from this definition. Objective measures such as pill counts or prescription refill records were not used.

### Statistical analysis

Changes in the proportion of patients with unused medications were analyzed using the McNemar’s test. Changes in DAI-10 scores were analyzed using the Wilcoxon signed-rank test. Statistical significance was set at *p* < 0.05. All analyses were performed using EZR version 1.52 (Saitama Medical Center, Jichi Medical University, Saitama, Japan).

### Ethical considerations

The study protocol was reviewed and approved by the Fukuoka Pharmaceutical Association Academic Ethics Review Committee (Approval No. 2023-003). All participants provided written informed consent prior to enrollment.

## Results

### Participants’ characteristics

A total of 52 patients were approached for participation; however, one patient declined due to time constraints and another was hospitalized before the second visit to our pharmacy, resulting in 50 patients being included in the final analysis.

Among the participants, 62% were women, and the mean age was 47.4 (SD, 13.7) years. All the participants were prescribed at least one antipsychotic medication. Specifically, 96% of the participants received second-generation antipsychotics, 28% received first-generation antipsychotics, and 58% were prescribed combined antipsychotics. Participant characteristics are described in Table 1.

**Table 1:** Patient characteristics (*n* = 50)

Characteristics	Number of participants (%)
(A) Sex, n (%)	
Female	31 (62.0)
Male	19 (38.0)
(B) Age (years)	
Mean (range)	47.4 (19–73 years)
Standard deviation	13.7
(C) Drugs, n (%)	
SGA	48 (96.0)
FGA	14 (28.0)
Combined administration of antipsychotics, n (%)	29 (58.0)
Duration of antipsychotic use, n (%)	
< 180 days	1 (2.0)
181–365 days	0 (0.0)
> 365 days	45 (90.0)
Unknown*	4 (8.0)
(D) Other drugs, n (%)	
BZP/Orexin receptor blockers/ramelteon	44 (88.0)
Antidepressants	19 (38.0)
Mood stabilizers	18 (36.0)
Anticholinergic drugs	12 (24.0)
Psychostimulants	4 (8)
(E) The times of taking pills per day**, n (%)	
1	5 (10.0)
2	6 (12.0)
3	19 (38)
4	19 (38)
5	1 (2)

*Due to insufficient information regarding medication use before visiting our pharmacy, the duration of medication use was unclear

** Daily dosing frequency was categorized as follows: once daily (each mealtime or bedtime), twice daily (e.g., morning and dinner time), three times daily (e.g., morning, dinner, and sleeping time), four times daily (e.g., every meal and sleeping time), five time (every meal time, at 21:00, and sleeping time)

SGA, second-generation antipsychotics; FGA, first-generation antipsychotics; BZP, benzodiazepines

All participants received antipsychotic treatment before enrolment in the study. Regarding the duration of antipsychotic use, 9.9% had been treated for less than 180 days, none (0%) had been treated for 180–365 days, and 90% had been treated for more than 365 days.

The participants were also prescribed additional psychotropic medications, including hypnotics (88%), antidepressants (38%), mood stabilizers (36%), anticholinergic agents (24%), and psychostimulants for attention-deficit/hyperactivity disorder (ADHD) (8%).

Medication was administered once daily by 10%, twice daily by 12%, three times daily by 38%, four times daily by 38%, and five times daily by 2% of the participants.

###  Improvement in attitude toward medication adherence (DAI-10 score)

As shown in Table 2, participants who did not use medication showed no significant improvement in their DAI-10 scores following pharmacist-led medication counseling (*p* = 0.748). In contrast, among participants who had unused medications, the DAI-10 scores improved significantly after the brief pharmacist-led intervention (mean increase, + 2.05 points; from 5.81 ± 4.00 to 7.86 ± 2.90, *p* = 0.00619).

**Table 2:** Change in DAI-10 in the normal and intervention groups (*n* = 50)

DAI-10 total score (Mean ± SD)	Before	After	*p*-value
Normal medication counseling (participants without unused drugs)	6.55 ± 3.11	6.90 ± 3.45	0.748
Pharmacist-led brief intervention (participants with unused drugs)	5.81 ± 4.00	7.86 ± 2.90	0.00619*

SD, standard deviation; DAI-10, Drug Attitude Inventory-10

*p < 0.05, Wilcoxon signed-rank test

### Questionnaire A: problems of medication adherence

42% of the participants had unused medications at the first assessment (Table 3). After the brief intervention, this percentage decreased significantly to 24% at the second assessment (*p* = 0.0159).

**Table 3:** Ratio of participants who reported non-adherence to medication regimens (*n* = 50)

Question	Response	Before (*n*)	After (*n*)	*p*-value
Do you ever forget to take your medication?		50	50	
Often or sometimes		21	12	0.0159*
Never		29	38	

n: number of participants. *p < 0.05, McNemar’s test

### Timing and reasons for missed doses

Before the intervention, the most commonly missed doses were in the morning (*n* = 13), lunchtime (*n* = 5), dinnertime (*n* = 9), and before sleeping (*n* = 4). After the intervention, these numbers decreased to 4 in the morning, 3 at lunchtime, 2 at dinnertime, and 1 before sleeping (Table 4).

**Table 4:** Questionnaire A before and after intervention in patients with problems at first assessment (*n* = 21)

Questions	Time of day	Before (*n*)	After (*n*)
When do you forget to take medication?	Morning Lunch time Dinner time Sleeping time	13 5 9 4	4 3 2 1
Patients with any of the following problems υ I tend to forget to take my medication for the following reasons:		21	
• I tend to skip meals		4	2
• I have difficulties coordinating medication timing with my daily activities		15	7
• I feel that I have to take medication too many times		3	0
• I sometimes get confused about how to take my medication		1	0
• I lose track of whether I have taken my medication		1	0
• I have no specific reason		1	0
υ I feel that I do not need to take medication for the following reasons:		3	0
• I take medication multiple times a day		2	0
• The number of pills I take at one time is high		2	0
• Taking medication is difficult (e.g., large size, capsules, unpleasant taste)		1	0
• I do not want others to see me taking medication		0	0
• I sometimes change how or how much medication I take depending on my mood		1	0
• Taking or not taking medication does not affect my body’s condition		0	0
• I do not understand what the medication is for or how the medication works		1	0
• I do not understand why I need to take the medication		0	0
• The medication makes me feel sick or causes side effects, such as:		2	0
o Tremor		1	0
o Gastric symptoms		1	0
o Weight gain		1	0

n: number of participants

Unintentional non-adherence was the most common reason for missed doses. Factors included skipping meals (*n* = 4 before the intervention; *n* = 2 after the intervention), difficulty coordinating medication timing with daily activities (before, *n* = 15; after, *n* = 7), feeling that the number of medications was excessive (before, *n* = 3; after, *n* = 0), confusion regarding medication use (before, *n* = 1; after, *n* = 0), and losing track of whether the medication had been taken (before, *n* = 1; after, *n* = 0).

Intentional non-adherence was also observed. Some participants specifically chose not to take medications because of difficulties in swallowing (*n* = 1 before intervention; *n* = 0 after intervention), changing the dosage regimen themselves (before, *n* = 1; after, *n* = 0), not feeling the effectiveness of the medication (before, *n* = 1; after = 0), or experiencing or worrying about adverse effects such as weight gain, tremors, or gastric discomfort (before, *n* = 2; after, *n* = 0).

In the second assessment, a few participants newly reported missed doses, primarily because of continued difficulties in coordinating medication timing (*n* = 4), perceiving that the number of medications was too high (*n* = 3), or tending to skip medication without a specific reason (*n* = 2). However, these newly emerging problems were relatively minor compared with the overall improvements.

## Discussion

Although the present study included patients prescribed antipsychotic medications regardless of a specific psychiatric diagnosis, previous studies have consistently reported high non-adherence rates among patients with major psychiatric disorders, ranging from 44 to 56% [17]. Consistent with these findings, our study identified a proportion of patients with adherence problems, with 42% reporting unused medication at baseline. These results indicate that medication non-adherence remains a persistent clinical challenge in the treatment of psychiatric disorders.

This study demonstrated that a brief pharmacist-led intervention conducted at a community pharmacy significantly improved medication adherence behaviors and attitudes among patients who were prescribed antipsychotic medications. Specifically, the intervention led to a substantial reduction in the proportion of patients taking unused medications and a significant improvement in the DAI-10 scores among those who initially experienced adherence problems. Both unintentional non-adherence, such as forgetfulness and difficulties in coordinating medication timing, and intentional non-adherence related to concerns about medication efficacy or adverse effects showed marked improvements after the intervention. These findings suggest that individualized pharmacist interventions can address a wide range of adherence barriers in patients with psychiatric disorders in community pharmacies.

Primary improvements were observed in cases of unintentional non-adherence, and preliminary indications of improvement were noted in a small number of patients exhibiting intentional non-adherence. Specifically, some patients who had previously modified their dosage regimens, discontinued medications due to perceived inefficacy, or expressed concerns about adverse effects, such as weight gain or tremors, appeared to show better adherence following the intervention. Although the sample size for intentional non-adherence was limited, these preliminary findings suggest that individualized pharmacist interventions may address both intentional and unintentional barriers to medication adherence.

However, it remains unclear why brief pharmacist-led interventions are effective in improving medication adherence. Previous research has indicated that poor insight into illness and negative attitudes toward medication are major factors contributing to non-adherence among patients with psychiatric disorders [18]. Negative experiences such as auditory hallucinations directed toward medication refusal, adverse events, suspicion about medication, and stigmatization by others (including families, neighbors, and community society) can induce non-adherence [17]. Addressing these psychological and cognitive barriers through tailored pharmacist interventions in community pharmacies may enhance trust in pharmacotherapy and reduce intentional medication avoidance.

Despite the overall improvements, the present results showed that a small proportion of participants developed new adherence problems in the second assessment. These issues, which appear to be related to changes in daily routine, lifestyle factors, and medication regimen adjustments, underscore the dynamic nature of patient adherence. The observation that new adherence problems have emerged over time suggests that while brief interventions can be effective, adherence support may need to be continuously adapted to patients’ changing circumstances.

From an implementation perspective, pharmacist-led interventions are feasible in routine community pharmacy practice. Counseling sessions per patient required approximately 10 min. No specialized certification was required. However, an experienced pharmacist who had good communication skills and experience in psychiatric pharmacotherapy was selected. Common barriers included limited consultation time during busy periods and physical limitations in some participants that limited independent self-completion of the questionnaires. However, these barriers were addressed with routine pharmacist support, and our intervention was generally well accepted without substantial additional resources.

The findings of this study are consistent with the international evidence supporting pharmacist-led adherence interventions. In countries such as the United States, the United Kingdom, and Europe, pharmacist-led adherence-supporting programs, such as medication reviews, educational interventions, and telemedicine, have demonstrated improvements in drug-related problems and medication adherence in mental health care [15]. Although health-care systems and reimbursement structures differ across countries, patient-centered counseling and systematic identification of adherence barriers are shared. Our results suggest that similar pharmacist-led approaches can be effectively applied in Japanese community pharmacy systems.

### Strengths and limitations

This study has several strengths. First, we objectively evaluated the effectiveness of a brief pharmacist-led intervention using validated measures, including the DAI-10, in a real-world outpatient setting. Second, the intervention targeted both intentional and unintentional non-adherence, offering a comprehensive understanding of the diverse barriers to adherence encountered by patients prescribed antipsychotic medications. Third, by tailoring the intervention based on individualized assessments, this study demonstrated the feasibility of integrating personalized adherence support into routine pharmacy practice.

However, this study has some limitations. This study was conducted at a community pharmacy in Japan with a relatively small sample size, which may limit the applicability of the findings to other healthcare systems. Additionally, the observational single-arm design, along with the absence of a control group and blinding, restricted the ability to infer causal relationships between the intervention and observed improvements. In addition, the Hawthorne effect may have contributed to improved adherence as participants were aware of being observed. Adherence was assessed using self-report measures that are inherently subject to social desirability and recall bias. Furthermore, the relatively short follow-up period prevented assessment of the long-term sustainability of the intervention effects. Given the dynamic nature of adherence behaviors, further studies are needed to examine factors such as dosing frequency and confirm these findings in long-term multicenter randomized controlled trials.

### Future directions

Future research should focus on evaluating the long-term effectiveness and sustainability of brief pharmacist-led interventions for medication adherence in patients with psychiatric disorders. Randomized controlled trials with larger and more diverse populations across multiple clinical sites are warranted to confirm the efficacy of such interventions and apply the findings to broader patient groups.

Moreover, research should aim to refine intervention components to optimize their impact. For example, identifying specific patient characteristics such as the level of illness insight, severity of symptoms, and presence of cognitive impairment to predict better responses to pharmacist-led interventions could enable more precise targeting of adherence support. Exploring the integration of technological support, such as mobile health applications, electronic reminders, and telepharmacy consultations, can also enhance the accessibility and effectiveness of pharmacist interventions.

Finally, future studies should investigate the clinical outcomes associated with improved adherence, such as reductions in relapse rates, hospitalization, and overall healthcare costs. The demonstration of tangible clinical and economic benefits would further strengthen the rationale for incorporating pharmacist-led adherence interventions into standard psychiatric care models.

## Conclusion

This study demonstrated that brief pharmacist-led interventions were effective in improving medication adherence, particularly by addressing unintentional non-adherence in patients receiving antipsychotic treatment. Although most patients benefited from the intervention, some required continuous support to maintain optimal adherence. Incorporating brief adherence counseling into routine pharmacy practice can enhance treatment outcomes in patients with psychiatric disorders. Future efforts for pharmacists should focus on developing sustainable, personalized, and multidisciplinary strategies to improve and maintain medication adherence over time.

## Data Availability

All data generated or analyzed during this study are included in this published article.

## Abbreviations

SGA: second-generation antipsychotics
FGA: first-generation antipsychotics
BZP: benzodiazepines
DAI-10: Drug Attitude Inventory-10
ADHD: attention-deficit/hyperactivity disorder

## References

[1] Ng R, El-Den S, Stewart V, Collins JC, Roennfeldt H, McMillan SS, et al. Pharmacist-led interventions for people living with severe and persistent mental illness: a systematic review. Aust N Z J Psychiatry. 2022;56:1080–103. 10.1177/00048674211048410. 34560826 10.1177/00048674211048410

[2] Emsley R, Chiliza B, Asmal L, Harvey BH. The nature of relapse in schizophrenia. BMC Psychiatry. 2013;13:50. 10.1186/1471-244x-13-50. 23394123 10.1186/1471-244X-13-50 PMC3599855

[3] Xiao J, Mi W, Li L, Shi Y, Zhang H. High relapse rate and poor medication adherence in the Chinese population with schizophrenia: results from an observational survey in the People’s Republic of China. Neuropsychiatr Dis Treat. 2015;11:1161–7. 10.2147/ndt.S72367. 26056450 10.2147/NDT.S72367 PMC4431492

[4] El Abdellati K, De Picker L, Morrens M. Antipsychotic treatment failure: a systematic review on risk factors and interventions for treatment adherence in psychosis. Frontiers in Neuroscience. 2020;14:531763. 10.3389/fnins.2020.531763. 33162877 10.3389/fnins.2020.531763 PMC7584050

[5] Shioda A, Yamauchi K. Community integration and related factors among people with mental illness in japan: multiple regression analysis stratified by social isolation level. Int J Soc Psychiatry. 2020;66:614–22. 10.1177/0020764020924690. 32475198 10.1177/0020764020924690

[6] Guerrero Z, Kågström A, Aliev A, Tomášková H, Yon Y, Lazeri L, et al. Mental health plans and policies across the WHO European region. Glob Ment Health (Camb). 2024;11:e110. 10.1017/gmh.2024.88. 39588205 10.1017/gmh.2024.88 PMC11588412

[7] Maehara M, Sugiyama M. Utilizing questionnaires for medication counselling of patients taking antipsychotics during the COVID-19 pandemic: a single site, community pharmacy-based survey study. J Pharm Health Care Sci. 2022;8:34. 10.1186/s40780-022-00263-w. 36474275 10.1186/s40780-022-00263-w PMC9727913

[8] Maehara M, Sugiyama M. A community pharmacist’s intervention in antipsychotic drug-induced sexual dysfunction in a patient with schizophrenia. Clin Case Rep. 2021;9:2074–6. 10.1002/ccr3.3946. 33936642 10.1002/ccr3.3946 PMC8077334

[9] Fujii T, Hanya M, Murotani K, Kamei H. Scale development and an educational program to reduce the stigma of schizophrenia among community pharmacists: a randomized controlled trial. BMC Psychiatry. 2021;21:211. 10.1186/s12888-021-03208-z. 33902519 10.1186/s12888-021-03208-z PMC8077925

[10] Black E, Murphy AL, Gardner DM. Community pharmacist services for people with mental illnesses: preferences, satisfaction, and stigma. Psychiatr Serv. 2009;60:1123–7. 10.1176/ps.2009.60.8.1123. 19648202 10.1176/ps.2009.60.8.1123

[11] Barkhof E, Meijer CJ, de Sonneville LM, Linszen DH, de Haan L. Interventions to improve adherence to antipsychotic medication in patients with schizophrenia—a review of the past decade. Eur Psychiatry. 2012;27:9–18. 10.1016/j.eurpsy.2011.02.005. 21561742 10.1016/j.eurpsy.2011.02.005

[12] Belvederi Murri M, Amore M. The Multiple Dimensions of Insight in Schizophrenia-Spectrum Disorders. Schizophr Bull. 2019;45:277–83. 10.1093/schbul/sby092. 29939361 10.1093/schbul/sby092 PMC6403083

[13] Schulze LN, Stentzel U, Leipert J, Schulte J, Langosch J, Freyberger HJ, et al. Improving medication adherence with telemedicine for adults with severe mental illness. Psychiatr Serv. 2019;70:225–8. 10.1176/appi.ps.201800286. 30651059 10.1176/appi.ps.201800286

[14] Tamaji A, Sakai A, Sato M, Ikema Y, Matsuzaki K, Tabuse H, et al. [Development of clinical pharmacy services to improve drug adherence in psychiatric hospital patients]. Yakugaku Zasshi. 2010;130:1565–72. 10.1248/yakushi.130.1565. 21048417 10.1248/yakushi.130.1565

[15] Ayre MJ, Lewis PJ, Keers RN. Understanding the medication safety challenges for patients with mental illness in primary care: a scoping review. BMC Psychiatry. 2023;23:417. 10.1186/s12888-023-04850-5. 37308835 10.1186/s12888-023-04850-5 PMC10258931

[16] Hogan TP, Awad AG, Eastwood R. A self-report scale predictive of drug compliance in schizophrenics: reliability and discriminative validity. Psychol Med. 1983;13:177–83. 10.1017/s0033291700050182. 6133297 10.1017/s0033291700050182

[17] Semahegn A, Torpey K, Manu A, Assefa N, Tesfaye G, Ankomah A. Psychotropic medication non-adherence and its associated factors among patients with major psychiatric disorders: a systematic review and meta-analysis. Syst Rev. 2020;9:17. 10.1186/s13643-020-1274-3. 31948489 10.1186/s13643-020-1274-3 PMC6966860

[18] Kim J, Ozzoude M, Nakajima S, Shah P, Caravaggio F, Iwata Y, et al. Insight and medication adherence in schizophrenia: an analysis of the CATIE trial. Neuropharmacology. 2020;168:107634. 10.1016/j.neuropharm.2019.05.011. 31077729 10.1016/j.neuropharm.2019.05.011

